# Behavioral and Neurophysiological Effects of Transdermal Rotigotine in Atypical Parkinsonism

**DOI:** 10.3389/fneur.2014.00085

**Published:** 2014-06-05

**Authors:** Davide Vito Moretti, Giuliano Binetti, Orazio Zanetti, Giovanni Battista Frisoni

**Affiliations:** ^1^IRCCS San Giovanni di Dio Fatebenefratelli, Brescia, Italy

**Keywords:** rotigotine, atypical parkinsonism, open-label study, safety, efficacy

## Abstract

Effective therapies for the so-called atypical parkinsonian syndrome (APS) such as multiple system atrophy (MSA), progressive supranuclear palsy (PSP), or corticobasal syndrome (CBS) are not available. Dopamine agonists (DA) are not often used in APS because of inefficacy and in a minority of case, their side effects, like dyskinesias, impairment of extrapyramidal symptoms or the appearance of psychosis, and REM sleep behavioral disorders (RBD). Transdermal rotigotine (RTG) is a non-ergot dopamine agonist indicated for use in early and advanced Parkinson’s disease with a good tolerability and safety. Moreover, its action on a wide range of dopamine receptors, D1, D2, D3, unlike other DA, could make it a good option in APS, where a massive dopamine cell loss is documented. In this pilot, observational open-label study we evaluate the efficacy and tolerability of RTG in patients affected by APS. Thirty-two subjects with diagnosis of APS were treated with transdermal RTG. APS diagnosis was: MSA parkinsonian type (MSA-P), MSA cerebellar type (MSA-C), PSP, and CBS. Patients were evaluated by UPDRS-III, neuropsychiatric inventory, mini mental state examination at baseline, and after 6, 12, and 18 months. The titration schedule was maintained very flexible, searching the major clinical effect and the minor possible adverse events (AEs) at each visit. AEs were recorded. APS patients treated with RTG show an overall decrease of UPDRS-III scores without increasing behavioral disturbances. Only three patients were dropped out of the study. Main AEs were hypotension, nausea, vomiting, drowsiness, and tachycardia. The electroencephalographic recording power spectra analysis shows a decrease of theta and an increase of low alpha power. In conclusion, transdermal RTG seems to be effective and well tolerated in APS patients.

## Introduction

Atypical parkinsonian syndromes (APS) comprise mostly progressive supranuclear palsy (PSP), corticobasal syndrome (CBS), and multiple system atrophy (MSA) (1–5). Despite decades of research, the cause and pathophysiology of atypical parkinsonian disorders are still unknown. While a poor response to dopamine agonists (DA) therapy is included as a criterion for MSA, PSP, and CBS, the literature suggests that about one-third of patients with each of the conditions does have a favorable response to the drug (6–8). A recent review (9) has demonstrated that APS patients experience a response to dopaminergic therapy that is generally modest and frequently transient. Also, all three conditions are similar because dopaminergic treatment rarely induces involuntary movements or mental status changes as well as RBD. Moreover, there have been reports of worsening motor function in patients with CBS, PSP, and MSA treated with dopaminergic therapy (9). As a consequence, therapeutic options are still limited.

Rotigotine (RTG) is a non-ergolinic dopamine agonist administered via a transdermal patch that delivers the drug over a 24-h period. (10). In six large, well designed clinical trials, RTG was an efficacious treatment for Parkinson’s disease, improving morning motor functioning, sleep disturbances, night-time motor symptoms, and depression. RTG was generally well tolerated across the trials and in longer-term extension studies (11–19). Thus, the RTG transdermal patch offers a novel therapeutical tool with efficacy in reducing disabling motor and non-motor symptoms, and acceptable tolerability profile.

To the best of our knowledge, transdermal RTG has been administered only in patients with idiopathic Parkinson’s disease but not in APS patients. Despite their different neuropathological features, MSA, PSP, and CBS are unanimously recognized as belonging to the nosographical category of the atypical parkinsonisms. Indeed, although MSA is an alpha-synucleinopathy and PSP/CBS are tauopathies, they share as final common pathway the degeneration of the dopaminergic system. The present observational trial has an eminently clinical approach. Indeed, the study was designed to provide a practical therapeutic option to neurologists and physicians in general when facing atypical parkinsonian syndromes. So, the further subdivision of the patients based on the neuropathological substrate was beyond the scope of the study. RTG could be a therapeutic option for APS because its mechanism of action encompasses a wide dopamine receptors spectrum (from D1 to D3), unlike other DA agents. Moreover, RTG patch could have the advantage of a minor adverse events (AEs) rate and the possibility to be administered in patients with swallowing problems. In the present study, RTG was administered to 32 patients with APS, showing an overall good performance on the motor symptoms, without inducing behavioral or extrapyramidal AEs.

## Materials and Methods

### Subjects

Thirty-two subjects with diagnosis of atypical parkinsonian disorders were admitted to our National Institute for Research and Cure of Neurodegenerative Disorders Fatebenefratelli (FBF) in Brescia and treated with transdermal RTG. Subjects were evaluated by DVM, a board-certified neurologist and movement disorders specialist who works in the Memory Clinic and Movement Disorders Center. All experimental protocols had been approved by the local ethics committee. Informed consent was obtained from all participants or their caregivers, according to the Code of Ethics of the World Medical Association (Declaration of Helsinki). Table 1 shows socio-demographical data of the whole study group.

**Table 1:** **Socio-demographical data of the whole study group; age and education are expressed in years**.

Subjects	MMSE	UPDRS/56	NPI/144	Age	Education
32	20.3	36.1	56.4	69.4	6.1

### Diagnostic criteria

In our subjects, diagnosis was as follows: MSA-P (11), MSA-C (4), PSP (7), and CBS (10). Moreover, all diagnoses were performed exclusively on clinical evaluation. Diagnostic criteria of atypical parkinsonism were inferred from the most diffuse guidelines and clinical criteria (20–30). Briefly, the classical PSP phenotype is characterized by postural instability and early falls, early cognitive dysfunction, and abnormalities of vertical gaze; it is referred to as Richardson’s Syndrome (RS). PSP was recently divided clinically and pathologically into two main phenotypes: classical PSP-RS and PSP-parkinsonism (PSP-P), the latter characterized by an asymmetric onset, tremor and moderate initial therapeutic response to levodopa (2, 29). In this study, the subjects affected by PSP were all classical PSP-RS type. The classical CBS phenotype consists of asymmetric parkinsonism, cortical signs (e.g., apraxia, cortical sensory loss, and alien limb), and possibly other signs such as dystonia and myoclonus; it is referred to as CBS. A recent study (29) proposed new criteria for probable and possible CBS. Probable CBS criteria require insidious onset and gradual progression for at least 1 year, age at onset ≥50 years, no similar family history or known tau mutations, and a clinical phenotype of CBS. The possible CBS category uses similar criteria but has no restrictions on age or family history, allows tau mutations, permits less rigorous phenotype fulfillment, and includes a PSP phenotype. Among our patients, seven met probable and three met possible CBS criteria. MSA is typically characterized by parkinsonism, autonomic dysfunction, and a combination of cerebellar and pyramidal signs. MSA is classified according to the predominant phenotype at onset into MSA-parkinsonism or (MSA-P), or MSA of the cerebellar type (MSA-C). Up to 80% of the patients develop most of the characteristic features during the course of the disease. The main concomitant pathologies of the patients in the study sample were: hypertension, hypercholesterolemia, hypertensive cardiopathy, diabetes mellitus type II, and carotid atheroma. As a consequence, patients were taking antihypertensive, antiplatelet, and antidiabetic drugs. The patients were encouraged to maintain as stable as possible the concomitant treatments. Patients who presented psychosis were not admitted to the study.

### Clinical evaluation

All patients underwent a complete physical and neurological examination. All patients underwent an electroencephalographic recording (EEG), and magnetic resonance imaging (MRI). Only 20 patients underwent single photon emission computed tomography with dopamine transporter scan (SPECT-dat scan). MRI was useful to exclude vascular parkinsonism, whereas SPECT shows diffuse and more symmetric degeneration of nigrostriatal pathways, excluding idiopathic Parkinson’s disease. Patients were assessed by the 56 points unified Parkinson’s disease rating scale (UPDRS) part III (31), neuropsychiatric inventory (NPI) (32), and mini mental state examination (MMSE) (33). More disease-specific scales are available to assess APS, like the Unified Multiple System Atrophy Rating Scale (UMSARS) (34) or the Progressive Supranuclear Palsy Rating Scale (PSPRS) (35) as well as the Scales for Outcomes in Parkinson’s disease-Autonomic (SCOPA-AUT) (36) or the movement disorders society modified UPDRS (MDS-UPDRS) (37). Anyway, the UPDRS scale has been chosen because it is well known and widespread. As a consequence, the general confidence of neurologists and physicians with this scale is very high. Moreover, it has the advantage that the results obtained are easily comparable with previous and future studies. Of note, a recent review (38) has convincingly demonstrated that the UPDRS is a reliable tool in the assessment of atypical parkinsonism. Evaluations were performed at baseline (T0) and during each follow-up. The follow-up visits were performed after 6, 12, and 18 months (T6, T12, and T18). The titration schedule was maintained very flexible, searching the major clinical effect and the minor possible AEs at each visit. When possible, the dosage was increased by 2 mg every 6 months. All AEs were recorded.

### EEG recordings

The EEG activity was recorded continuously from 19 sites by using electrodes set in an elastic cap (Electro-Cap International, Inc.) and positioned according to the 10–20 international systems (Fp1, Fp2, F7, F3, Fz, F4, F8, T3, C3, Cz, C4, T4, T5, P3, Pz, P4, T6, O1, and O2). In order to keep constant the level of vigilance, an operator controlled on-line the subject and the EEG traces, alerting the subject any time there were signs of behavioral and/or EEG drowsiness. The ground electrode was placed in front of Fz. The left and right mastoids served as reference for all electrodes. The recordings were used off-line to re-reference the scalp recordings to the common average. Re-referencing was done prior to the EEG artifact detection and analysis. Data were recorded with a band-pass filter of 0.3–70 Hz, and digitized at a sampling rate of 250 Hz (BrainAmp, Brain Products, Germany). Electrodes-skin impedance was set below 5 kHz. Horizontal and vertical eye movements were detected by recording the electrooculogram (EOG). The recording lasted 5 min, with subjects with closed eyes. Longer recordings would have reduced the variability of the data, but they would also have increased the possibility of slowing of EEG oscillations due to reduced vigilance and arousal. EEG data were then analyzed and fragmented off-line in consecutive epochs of 2 s, with a frequency resolution of 0.5 Hz. The average number of epochs analyzed was 140, ranging from 130 to 150. The epochs with ocular, muscular, and other types of artifacts were discarded by two skilled electroencephalographists (39). EEG recordings were performed at baseline as well as at each of the follow-up control visits.

### Analysis of individual frequency bands

All recordings were obtained in the morning with subjects resting comfortably. Vigilance was continuously monitored in order to avoid drowsiness. A digital FFT-based power spectrum analysis (Welch technique, Hanning windowing function, no phase shift) computed – ranging from 2 to 45 Hz – the power density of EEG rhythms with a 0.5 Hz frequency resolution. Two anchor frequencies were selected according to the literature guidelines, that is, the theta/alpha transition frequency (TF) and the individual alpha frequency (IAF) peak. These anchor frequencies were computed on the power spectra averaged across all recording electrodes. The TF marks the TF between the theta and alpha bands, and represents an estimate of the frequency at which the theta and alpha spectra intersect. TF was computed as the minimum power in the alpha frequency range, since our EEG recordings were performed at rest. The IAF represents the frequency with the maximum power peak within the extended alpha range (5–14 Hz). Based on TF and IAF, we estimated the frequency band range for each subject, as follows: delta from TF-4 to TF- 2, theta from TF-2 to TF, low alpha band from TF to IAF, and high alpha band from IAF to the point of the minimum power after the IAF values. The mean frequency range computed in MCI subjects considered as a whole are: delta 2.9–4.9 Hz; theta 4.9–6.9 Hz; low alpha1 6.9–10.9 Hz; and high alpha2 8.9–11.9 Hz. Finally, in the frequency bands determined on an individual basis, we computed the relative power spectra for each subject. The relative power density for each frequency band was computed as the ratio between the absolute power and the mean power spectra from 2 to 45 Hz. The relative band power at each band was defined as the mean of the relative band power for each frequency bin within that band (39).

### Statistical analysis

One-way analysis of variance (ANOVA), with Newman–Keuls *post hoc* correction, was performed to test the changes in MMSE, UPDRS-III, and NPI considering all the evaluations (T0, T6, T12, and T18). Also analysis of EEG results has been performed with ANOVA.

## Results

Table 2 summarizes the main results.

**Table 2:** **Number of subjects and clinical characteristics at baseline and T6, T12, and T18 follow-up (between the brackets the number of subjects dropped out)**.

	T0	T6	T12	T18
Subjects	32	29 (3)	20 (0)	13 (0)
MMSE	20.3	19.3	18.1	15.3
UPDRS	36.1	28.6	26.5	25.5
NPI	56.4	50.1	41.2	35.9
RTG dosage	n.d.	3.2	3.7	4.4

At baseline, mean UPDRS-III score was 36.1/56, mean NPI score was 56.4/144, mean MMSE score was 20.3/30. 29/32 (90.7%) patients has reached 6 months of treatment with RTG (mean dose = 3.2 mg/24 h); mean UPDRS-III score was 28.6/56, mean NPI score 50.1/144, mean MMSE score 19.3/30. Three patients were dropped out for AEs. 23/32 (71.8%) patients has reached 12 months of treatment with RTG (mean dose = 3.7 mg/24 h), mean UPDRS-III score was 26.5/56, mean NPI score was 41.2/144, mean MMSE score was 18.1/30. No patient more was dropped out for AEs at 12 months follow-up. 16/32 (50%) patients completed 18 months of treatment with RTG (mean dose = 4.4 mg/24 h) mean UPDRS-III score was 25.5/56, mean NPI score was 35.9/144, mean MMSE score was 15.3/30. No patient was dropped out at 18 months follow-up (Table 2). Reported AEs were: hypotension (five patients), nausea (four), vomiting (three), drowsiness (two), and tachycardia (one). On the whole, 15 patients were affected by transient AEs (46.8%) and only 3 patients suspended RTG treatment due to AE (9.3%; vomiting, tachycardia, and sleepiness). No heart congestion failure was detected among our patients. All other patients are yet in the study, although with different follow-up.

**Figure 1:**
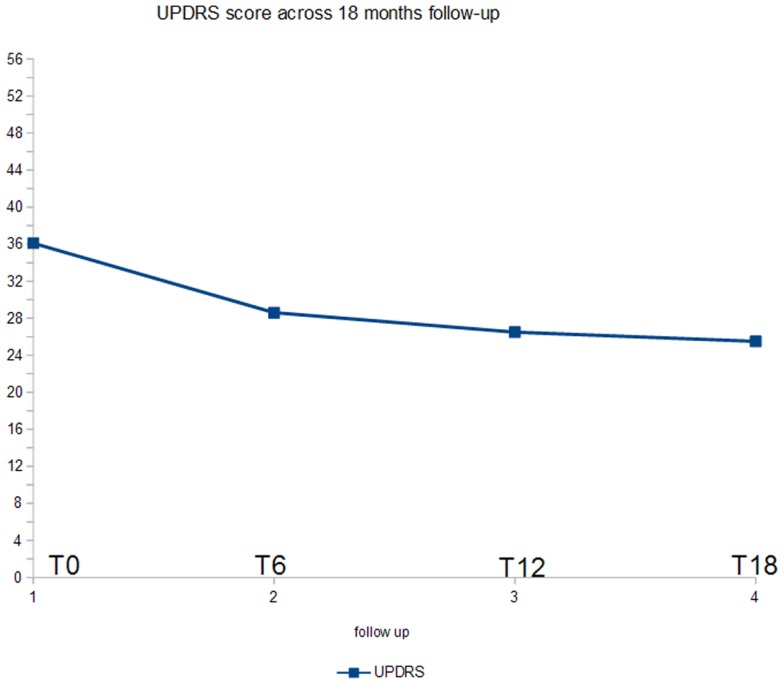
**ANOVA results for UPDRS score**.

Figure 1 shows the ANOVA results for UPDRS-III. A significant decrease of the score (*p* < 0.005) was found. In *post hoc* analysis, all the comparisons with the baseline were statistically significant (*p* < 0.02).

**Figure 2:**
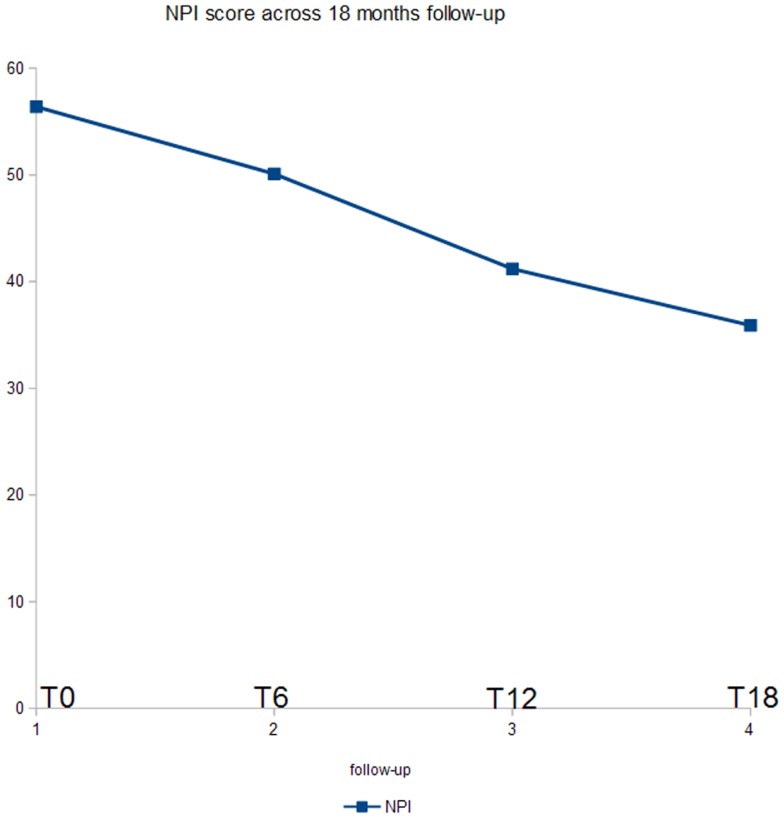
**ANOVA results for NPI score**.

Figure 2 shows the ANOVA results for NPI. A significant decrease of the score (*p* < 0.03) was found. In *post hoc* analysis, all the comparisons with the T18 follow-up were statistically significant (*p* < 0.04). Moreover, the evaluation at T12 was significant as compared to the baseline (*p* < 0.05).

**Figure 3:**
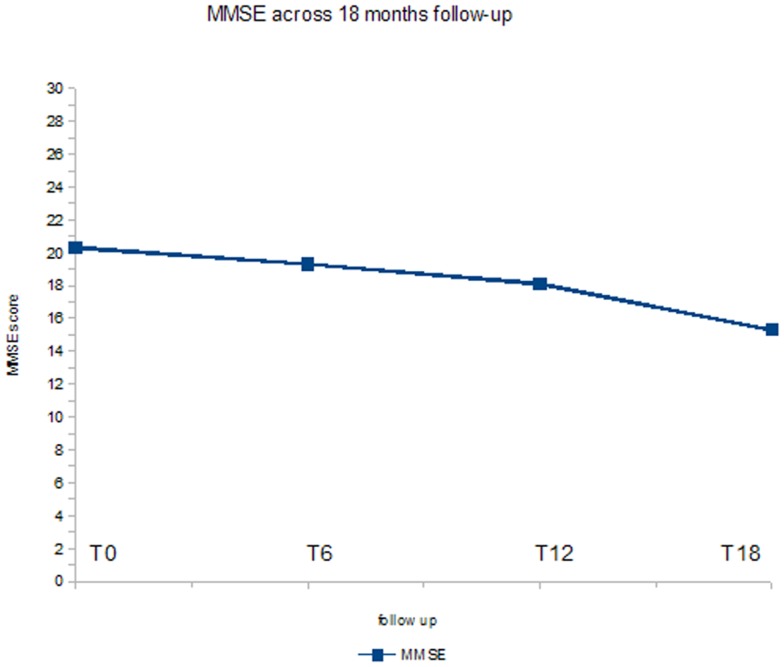
**ANOVA results for MMSE score**.

Figure 3 shows the ANOVA results for MMSE. A significant decrease of the score (*p* < 0.05) was found. In *post hoc* analysis, the comparison between baseline evaluation and the T18 follow-up was statistically significant (*p* < 0.01).

The power spectra show a significant decrease of theta and increase of low alpha band but no for high alpha power. These results were present at the 6-month (theta *p* < 0.001, low alpha *p* < 0.002, high alpha *p* = 0.09) follow-up and were maintained at both the 12-month follow-up (theta *p* < 0.002, low alpha *p* < 0.001, high alpha *p* = 0.08) and the 18-month follow-up (theta *p* < 0.004, low alpha *p* < 0.005, high alpha *p* = 0.09).

## Discussion

### Preliminary remarks

From an heuristic point of view, a more focused analysis, splitting the groups for single pathology, or including at least the separation of MSA from PSP and CBS, would be methodologically correct. Anyway, it is well known that, in the early stage of the disease, the differentiation of the various forms of atypical parkinsonian disorders can be challenging. In some cases, PSP presents with features of CBS, including apraxia, alien limb phenomena, and cortical sensory loss (40). Most PSP, but also some MSA cases, present with early falls and supranuclear vertical gaze palsy (26). Early autonomic dysfunctions including urinary urgency, frequency or nocturia without hesitancy, chronic constipation, postural hypotension, sweating abnormalities, and erectile dysfunction are reasonable discriminators of MSA but could be present in PSP or CBS (3, 41–44). As a consequence, the first need of neurologists and physicians in the clinical practice is to find a safe and possibly efficacious treatment for atypical parkinsonisms considered as a whole. Nonetheless, we claim that further studies will assess the remaining controversial aspects in APS diagnosis to find targeted therapies according to the neuropathological alterations. In the present study, we try to provide a suitable treatment option in a very poor therapeutic context.

### Current treatment options in atypical parkinsonian syndrome

Although there are still no treatments available for the sporadic atypical parkinsonian conditions, important efforts have been done in recent years, which, even if not proven effective clinically, will certainly guide further research. A randomized, placebo-controlled clinical trial to assess the effects of treatment with the monoamine oxidase-B inhibitor rasagiline (1 mg/day) for 48 weeks in 174 patients with possible or probable MSA-Parkinsonism type, in 39 sites in 12 countries, found no significant difference in progression in the total UMSARS score between the verum and placebo groups (45). A single-arm, single-center, open-label pilot trial evaluated monthly infusions of 0.4 g/kg intravenous immunoglobulin for 6 months in seven patients as an anti-inflammatory approach, and found significantly improved UMSARS part I (activities of daily living) and II (motor functions); verification in a controlled study was proposed (46). A recent study compared 30–50 intraarterial or intravenous injections of autologous mesenchymal stem cells (MSCs) vs. placebo in 33 patients with probable MSA-cerebellar type and suggested that the MSC group had a smaller increase in total and part II UMSARS scores from baseline throughout a 360-day follow-up period; as the mechanism of action of this intervention remains unclear, a careful experimental and clinical re-evaluation of these findings should be considered (47).

In regard to PSP, a multinational phase 2/3 randomized, double-blind, placebo-controlled trial enrolled 313 participants, to be treated with 30 mg davunetide or placebo twice daily for 52 weeks at 47 sites, and found no significant effect on the co-primary outcome measures, the PSPRS and the Schwab and England Activities of Daily Living (SEADL) (Press release December 18, 2012 by Allon Therapeutics, www.allontherapeutics.com). An open-label pilot trial of lithium, an inhibitor of glycogen synthase kinase-3 (GSK-3), in individuals with PSP or CBS (ClinicalTrials.gov Identifier NCT00703677) recruited 17 patients and was stopped prematurely because the majority of participants did not tolerate the study drug. A multinational, phase II, double-blind, placebo-controlled trial enrolled 142 patients with PSP, who were treated orally with tideglusib (600 or 800 mg p.d.), also a GSK-3 inhibitor, or placebo for 1 year. There were no significant differences between the high dose, low dose, and either dose groups vs. the placebo group in the primary clinical outcome measures. A subset of 37 patients underwent baseline and 52-week MRI; this substudy demonstrated significantly reduced global brain atrophy in tideglusib-treated patients (48). The effect of GSK-3 inhibition in PSP thus warrants further investigation (49).

### Transdermal RTG as treatment option for atypical parkinsonian syndrome

Transdermal RTG seems to be effective and well tolerated in patients with APS. Our results show significant improvement in UDPRS-III scores, maintained along the course of the 18 months follow-up. Moreover, only 3 patients were dropped out and 15 patients were affected by transient AEs. A plausible explanation of the results is that RTG transdermal patch has a wider dopaminergic profile action as compared to other non-ergot, prolonged-release DA, allowing a more diffused reply therapy, covering the impotent dopaminergic loss in atypical parkinsonism (50). Indeed, RTG has a dopaminergic agonist action on D1, D2, D3 dopamine receptors, unlike pramipexole (D3 agonist) and ropinirole (D2/D3 agonist). Our results show also a reduction of NPI scores, which became significant at the T12 and T18 follow-up evaluation. Previously, it has been demonstrated that RTG was efficacious in reducing sleep disturbance and other non-motor symptoms in PD patients (51, 52). This safety profile could be explained by the particular mechanism of action of RTG. RTG is a non-ergolinic dopamine agonist with direct actions at dopamine receptors (D1–3) (53). Of note, RTG has its highest affinity for and activity at D3 receptors (53). D3 receptors are sparse in the caudate–putamen region, but densely populated in ventral striatum and appear to play a modulatory role on motor output and the affective state (53). RTG also had affinity to non-dopaminergic receptors, such as α2B-adrenergic receptors and serotonin 5-HT1A receptors that could positively modulate mood and behavior. The duration of the therapeutic effect on both motor and behavioral symptoms until 18 months of follow-up could suggest a neuroprotective effect. Although previously seen in animal models and *in vitro* studies (54), this aspect needs to be cautious and further assessments. During the study, our patients did not suffer from congestive heart failure. Of note, RTG had low affinity for serotonin 5-HT2B receptors which may be of clinical importance, as ergolinic DA thought to cause cardiac valvular damage are full or partial serotonin 5-HT2B receptor agonists (55, 56).

Our results show no positive effect on cognitive status. Anyway, the MMSE score shows that patients were highly cognitively impaired, on average, at the beginning of the study. Further studies, with less initially cognitively compromised patients and grouped for single pathologies, will better clarify this issue.

### EEG oscillatory activity

Our results confirm previous works showing that the most significant finding in Parkinson’s disease synchronized oscillatory activity is the prevalence of a rhythmic activity in the range of 4–7 Hz, usually termed theta band. This activity is characteristic of the basal ganglia-thalamocortical circuits, and such activity can be reduced by dopaminergic treatments (57). The increase of the low alpha activity could be explained by the relief of large thalamo-cortical circuits, in particular the loop involving higher order brain areas, typically entrained by the alpha rhythm. On the contrary, the lack of effect on high alpha oscillatory activity could suggest a poor effect on cortico-cortical connections, which gave their contribution on the upper alpha rhythm component.

### Study limitations

This study is a pilot, open-label, observational study with a clear explorative purpose. First of all, we have to remark that small sample size has not made possible the investigation on the single pathology level because of the lack of any statistical power. Of note, the results could not be extended to other conditions such as the primary progressive freezing of gait preceding the PSP, because we have not investigated such phenotype. As a consequence, further randomized, double-blind, placebo-controlled trial studies with a bigger sample size and with more disease-specific scales and assessment for both the daily living functionality and the statistical evaluation are needed to confirm the present results. Anyway, the favorable outcomes could be very useful as a practical guideline to help clinicians and neurologists to find a treatment in atypical parkinsonisms.

## Conclusion

In our observational, open-label study, RTG appears to be a suitable therapy in APS patients as it has a good tolerability and efficacy profile. The activation of D1–D2–D3 receptor in the caudate–putamen region by RTG patch, compensates for the spread loss of dopaminergic function in these areas and could be responsible for the efficacy of this drug. Anyway, more studies are mandatory to confirm the results.

## Author Note

Davide Vito Moretti: on the behalf of all coauthors I declare that appropriate approval and procedures were used concerning human subjects.

## Conflict of Interest Statement

The authors declare that the research was conducted in the absence of any commercial or financial relationships that could be construed as a potential conflict of interest.
